# Editorial: Ethical design of artificial intelligence-based systems for decision making

**DOI:** 10.3389/frai.2023.1250209

**Published:** 2023-07-24

**Authors:** Giulio Biondi, Stefano Cagnoni, Roberto Capobianco, Valentina Franzoni, Francesca A. Lisi, Alfredo Milani, Jordi Vallverdú

**Affiliations:** ^1^EmoRe Research Group, Department of Mathematics and Computer Science, University of Perugia, Perugia, Italy; ^2^Department of Engineering and Architecture, University of Parma, Parma, Italy; ^3^Artificial Intelligence and Robotics Research Group, Department of Computer, Control and Management Engineering, La Sapienza University of Rome, Rome, Italy; ^4^Department of Computer Science, Hong Kong Baptist University, Kowloon, Hong Kong SAR, China; ^5^Department of Computer Science, University of Bari “Aldo Moro”, Bari, Italy; ^6^ICREA Acadèmia, Department of Philosophy, Universitat Autònoma de Barcelona, Barcelona, Catalonia, Spain

**Keywords:** ethics, artificial intelligence, decision support, AI regulation, ethics in AI, fairness

## Introduction

Emphasizing the importance of ethical design in AI-based decision-making systems is not only crucial from an emotional and social perspective but also from a legal and risk management standpoint (see Crawford and Calo, 2016). While EU regulations, such as Madiega (2021) or European Commission (2019), impact all Artificial Intelligence (AI) products in European countries, it is important to note that in the United States, AI regulations are voluntary and locally applied. On January 26, 2023, the National Institute of Standards and Technology (NIST), an agency of the US Department of Commerce, released the Artificial Intelligence Risk Management Framework 1.0 (RMF) (see Tabassi, 2023). This framework serves as a voluntary, non-sector-specific guide for technology companies engaged in the design, development, deployment, or utilization of AI systems. Its objective is to assist these companies in effectively managing the diverse risks associated with AI. AI technologies are subject to various legal frameworks and regulations that govern their use and mitigate potential risks. Ethical design ensures that AI systems comply with legal requirements, such as data privacy and protection laws, but also with human psychological and emotional needs (Vallverdú and Casacuberta, 2014; Franzoni and Milani, 2019); it incorporates mechanisms to safeguard personal information and ensure that AI systems operate within the bounds of legal frameworks (Coeckelbergh, 2020). Furthermore, ethical design considers risk management in the development and deployment of AI systems. It involves identifying and assessing potential risks associated with biases, discrimination, or unintended consequences (Buolamwini and Gebru, 2018; Biondi et al, 2022). By integrating risk management practices, such as rigorous testing, validation, and ongoing monitoring, the ethical design minimizes the likelihood of negative outcomes and helps mitigate legal liabilities, in both local and global domains (Jobin et al., 2019). On June 20th, 2023, the European Parliament made significant progress in shaping the AI Act by adopting its negotiating position. This move aims to ensure that AI systems developed and utilized in Europe adhere to the principles and values of the European Union (EU), including human oversight, safety, privacy, transparency, non-discrimination, and social and environmental wellbeing. The Parliament's position highlights several key aspects. Firstly, they advocate for a complete ban on the use of AI for biometric surveillance, emotion recognition, and predictive policing. Secondly, they propose that generative AI systems, such as ChatGPT, should clearly disclose when content is AI-generated. Lastly, the Parliament considers AI systems used for influencing voters in elections as high-risk. The ethical design can also align with ethical guidelines and principles set forth by professional and regulatory bodies. Adhering to these guidelines promotes responsible and accountable use of AI technologies, reducing legal risks and ensuring compliance with industry standards. In summary, ethical design in AI-based decision-making systems goes hand in hand with legal compliance and risk management. It ensures that AI systems are developed and operated within legal boundaries, while also minimizing risks and liabilities. By embracing ethical principles, organizations can navigate the complex legal landscape surrounding AI technologies and mitigate potential legal and reputational risks associated with their deployment (see Vinuesa et al., 2020; Franzoni, 2023). By incorporating ethical considerations, AI-based decision-making systems can avoid perpetuating biases, discrimination, and other negative social consequences (see Biondi et al., 2022). Ethical design takes into account the diverse needs, preferences, and emotions of individuals, promoting inclusivity and fairness (Zafar et al., 2017). It recognizes the importance of transparency and interpretability, enabling users to understand and trust the decisions made by AI systems. Moreover, ethical design acknowledges the potential impact of AI decisions on social dynamics and relationships. It encourages responsible behavior and accountability, ensuring that AI systems are designed to align with societal norms and values. By prioritizing ethical design, we can ensure that AI technologies contribute positively to society while respecting the emotional and social fabric of human existence.

## State of the art

Ethical design in AI-based decision-making systems is of paramount importance. Current approaches, methodologies, and frameworks address the ethical implications associated with these technologies. There are some fundamentals to be taken into account: Integration of fairness and non-discrimination principles promotes equitable outcomes and mitigates bias (Floridi et al., 2020); transparency and interpretability enhance trust and accountability (Larsson and Heintz, 2020); accountability ensures clear responsibility and mechanisms for addressing potential harms (Mittelstadt, 2019); privacy preservation techniques safeguard sensitive data while enabling collaboration (Manheim and Kaplan, 2019); and, finally, the ethical design fosters trust in AI technologies and mitigates unintended consequences (Bryson and Winfield, 2017). Challenges include balancing fairness and accuracy and addressing interpretability-performance trade-offs. Of course, practical and scalable frameworks are needed. Emphasizing ethical design in AI-based decision-making systems addresses societal concerns, reduces biases, enhances transparency, and establishes accountability (Novelli et al., 2023). Ongoing analysis promotes responsible AI systems aligned with societal values, benefiting individuals and communities. Therefore, exploring current approaches, methodologies, and frameworks in ethical design for AI systems is essential in addressing the ethical challenges posed by AI technologies. Researchers and practitioners have made significant strides in developing strategies to ensure responsible and accountable AI systems.

## Research Topic on ethical design of artificial intelligence-based systems for decision making

### Systematic reviews

In virtual educational settings, the impact of learner and teacher gender on human-to-human interaction and the persistence of gender stereotypes are of critical interest. In the systematic review of studies on Pedagogical Agents by Armando et al., authors discuss the impact of gender on learners' perception, academic performance, and self-evaluation skills. Findings indicate that male and female agents can improve performance, with female agents efficiently employable to contrast the stereotype threat, e.g., in male-dominated STEM environments. On the other hand, the agents' gender evidently impacts their pedagogical roles, appearance, and interaction patterns. Virtual agents whose gender does not match social stereotypes on context and roles may be less effective in conveying their messages e.g., older and elegant agents are perceived as experts; female agents are more successful in establishing positive relationships with learners. Androgynous systems as a potential solution require further investigation, as they may hinder efforts to avoid gender stereotypes. The review emphasizes the importance of gender choice and the need for further research in this area.

In the field of green economy and, in particular, regarding waste management applications, the review by Nkwo et al. highlights the significance of thoughtful and human-centered design in developing applications that raise awareness of social issues, using the Persuasive System Design (PSD) framework. The study investigates the incorporation and implementation of behavior change strategies and evaluates their effectiveness based on user ratings. The findings reveal that task-assistance strategies are prevalent, while credibility strategies enhance persuasiveness and trust. The impact of dialogue support strategies, feedback and reminder provisions, and social support strategies leveraging social influence across various dimensions, including app focus and waste management activities, correlate with app ratings. Based on the findings, the authors provide design suggestions and guidelines leveraging social influence e.g., sustainable waste management apps, emphasizing user-friendly routines, adaptive features, automated intelligent notifications, and performance tracking.

### Novel research contributions

The three original research papers in this Research Topic (i.e., Thomas et al.; Chen et al.; Wang et al.) present contrasting viewpoints on user experience with digital interactive systems. Two papers analyze user behavior, while the third examines the impact of messages conveyed through such systems.

In Thomas et al., the authors critically review existing approaches to assessing message persuasiveness in different domains. As a result of their analysis, the authors propose and validate a new scale of persuasiveness based on user ratings of items from two domains: healthy eating advice and email security messages.

The other two papers focus on monitoring literacy learners' attention status and users' attitudes toward medical Artificial Intelligence. In Chen et al., the authors introduce a method to assess disengagement among literacy learners during online classes by measuring performance discrepancy between control tests proposed during class and pre-class tests proposed at the very beginning of the class (i.e., when students' attention is expected to be optimal). The authors show a strong correlation between high attention ratings obtained through their method and good performance in post-test reading comprehension.

In Wang et al., the authors examine methods for assessing people's Knowledge, Attitude, and Behavior (KAB) regarding medical AI. In doing so, they compare a person-based approach that stratifies a population's KAB based on individual profiles with the more common variable-based approach relying on isolated self-assessments of each component. This approach highlights the emergence of subtler profiles of interaction among the three components.

Overall, these papers provide valuable insights into understanding user experience, attention, and attitudes in AI interactive systems, offering new scales, assessment methods, and approaches for further exploration.

### Opinion and perspective contributions

Since AI systems are increasingly relied upon for decision-making across different domains, limitations and risks associated with certain applications of AI need to be taken into consideration. Nathan and Fourneret and Yvert aim to shed light on critical issues associated with the use of AI systems.

Nathan (2023) focus on the limitations of disembodied AI (dAI) in educational systems, which emerged particularly during the COVID-19 pandemic. Such systems have two significant limitations: they struggle to model people's embodied interactions, as they primarily rely on statistical regularities rather than capturing the nuanced nature of human behavior; and they are often black boxes, lacking transparency and predictability when applied to new domains. The emergence of multimodal learning analytics and data mining (MMLA) exacerbates the issue, as data accessibility and usage are not properly regulated. To mitigate the risks associated with dAI, Nathan proposes an alternative augmented intelligence system that effectively addresses students' needs.

On the other hand, Fourneret and Yvert highlight a more subtle risk associated with using AI systems to aid human decision-making: human desubjectivation. People's increasing reliance on AI system recommendations has led to various forms of digital normativity, where algorithms establish standards that individuals adopt as the norm in their daily lives, a phenomenon that may affect the acquisition and exercise of subjectivity, influencing critical thinking. Relying entirely on AI systems for decision-making promotes human comfort but discourages individuals from challenging or refusing system suggestions due to their perceived infallibility. To address the risk of desubjectivation, Fourneret and Yvert highlight the importance of an Ethics-by-design methodology, involving ways to protect the subjective thinking process during the project's ideation phase rather than at implementation. They emphasize the importance of involving philosophers and ethicists in the development of new technologies and emphasize the need to educate future generations about the risks of silent acceptance of AI governmentality (see also Franzoni, 2023).

## Open problems and future work

Despite the ongoing debates and discussions regarding the ethical aspects of AI, practical solutions to ensure shared ethics remain open challenges.

### Transparency and explainability

One of the significant challenges lies in the transparency and explainability of AI systems. Generalist AI systems often employ sophisticated algorithms and deep neural networks, making it difficult to understand and explain their decision-making processes (see Adadi and Berrada, 2018; Balasubramaniam et al., 2023). The lack of transparency and interpretability raises concerns about discrimination and unfair or unjust outcomes.

### Accountability and responsibility, autonomy, human oversight, and control

As AI systems take on increasingly autonomous decision-making roles, traditional models of responsibility may not adequately capture the new unique challenges posed. Establishing clear frameworks for assigning responsibility and addressing questions of negligence, oversight, and the potential for unintended consequences is essential to ensure accountability for the decisions made by AI systems, capable of making autonomous decisions across various domains without human intervention. Balancing the autonomy of AI systems with human judgment and intervention is necessary to prevent undue reliance on AI decisions and preserve human agency and accountability (see Beckers, 2022; Cavalcante Siebert and Lupetti, 2023).

### Bias and fairness, Societal impact, and distribution of benefits

AI systems can inadvertently perpetuate biases present in the data they are trained on, leading to discriminatory outcomes (see Dwork, 2012; Mehrabi, 2021). Decision-making AI must be designed to recognize and mitigate biases, ensuring fairness in the decision-making process across diverse populations. This issue requires developing techniques that identify and address biases and allow designers to be conscious of their biases and limits. AI systems can have significant societal impacts, influencing resource allocation, access to services, and opportunities. Ensuring these systems are designed and deployed to benefit all individuals is critical to avoid exacerbating existing inequalities (Datta, 2023).

### Privacy and data security

Generalist AI systems rely on vast amounts of data, often including sensitive personal information. Protecting individual privacy and ensuring robust data security measures become paramount to prevent misuse or unauthorized access to personal information. Balancing the benefits of artificial intelligence with privacy considerations is an ongoing challenge, as a huge number of entities are massively and continuously collecting data, virtually beyond the control of individuals (see Song et al., 2022; USA White House Executive Office, 2023).

## Conclusion

In the new era of Generalist AI, where AI systems are expected to handle a wide range of tasks and exhibit human-like capabilities, the challenges, and complexities of ethical design will become more pronounced. AI systems may encounter situations where ethical dilemmas arise, such as conflicts between different moral values or competing interests (see Xiaoling, 2021; Huang et al., 2022). Deciding how to prioritize and navigate these ethical dilemmas becomes crucial. Establishing clear ethical frameworks and guidelines to make ethically sound decisions is a complex challenge (see Ramos and Koukku-Ronde, 2022; UNESCO, 2022). Researchers must explore interdisciplinary collaborations that combine expertise in AI, ethics, philosophy, law, and social sciences. This collaborative approach can pave the way for developing comprehensive ethical frameworks, and standards that govern the design, deployment, and use of AI-based decision-making systems.

## Author contributions

VF and JV: conception and design of the work. JV: draft—Sections Introduction and State of the art. GB: draft—Section Systematic reviews. SC: draft—Section Novel research contributions. RC: draft—Section Opinion and perspective contributions. AM: draft—Sections Open problems and future work, Conclusion. VF and FL: critical revision of the manuscript. VF: work supervision and review. All authors provide approval for publication of the content and agree to be accountable for all aspects of the work, ensuring that questions related to the accuracy or integrity of any part of the work are appropriately investigated and resolved.
